# Supplementary material for: Are carbon emissions trading and green financial instruments synergistic? -Comprehensive quantitative research based on content analysis
Source: PLoS One. 2024 Mar 7;19(3):e0298601. doi: 10.1371/journal.pone.0298601 (PMC10919601; doi:10.1371/journal.pone.0298601)
Supplement: S1 Data — (ZIP) [file pone.0298601.s001.zip › Data/地级市Z_F工作报告.zip!/╣π╢1⁄2/│▒╓▌╩╨/└┤╫╘╣1⁄2╓┌║┼í╛┬φ┐╦╩2╛▌═°í┐_2008.docx]

各位代表：

我代表市人民政府向大会作工作报告，请予审议，并请政协各位委员和其他列席人员提出意见。

2007年工作回顾

2007年，是本届政府实现良好开局的一年。过去一年，市政府在省委、省政府和市委的领导下，在市人大及其常委会和市政协的监督支持下，高举中国特色社会主义伟大旗帜，以邓小平理论和“三个代表”重要思想为指导，全面落实科学发展观，认真贯彻党的十七大、省第十次党代会和市第十二次党代会精神，按照“三年打基础、五年大变化、十年大发展”的要求，围绕建设“一名城两基地”目标，带领全市人民奋力推进经济社会又好又快发展，圆满完成了市十三届人大一次会议确定的各项任务。

一、经济综合实力进一步增强

经济增长速度加快。全市生产总值387.5亿元，增长14.2%，比上年提高1.2个百分点，接近全省平均水平，增速为近10年来最快，在全省21个地级以上市的排名由上年的第18位上升至第14位。人均生产总值15256元，增长13.6%。一、二、三产业分别增长4.1%、15.3%和15.1%，三大产业结构由上年的9.5 ：56.2 ：34.3调整为9.1 ：56.4 ：34.5。

经济运行质量提高。地方财政一般预算收入13.5亿元，增长26.8%。各项税收收入38.42亿元，增长29%。其中，国税国内税收25.66亿元，地税税收12.36亿元，分别增长34.7%和19.3%。国有资产监管力度加大，劣势企业退市步伐加快。规模以上工业企业盈利能力增强，实现利税33.2亿元，增长62.5%，比上年提高4.5个百分点；经济效益综合指数154.4%，比上年提高19.5个百分点。金融支持力度加大，年末本外币各项存款余额393.8亿元、贷款余额160.1亿元，分别增长5.2%和16.4%，存贷比例由上年的36.7%提高至40.7%。

固定资产投资增长。全社会固定资产投资121.04亿元，增长10.3%。其中，更新改造投资36.34亿元，增长11.2%；工业投资58.85亿元，增长9.8%。年度47个重点建设项目完成投资39.57亿元，占计划的111.2%。省道335线潮州段改建工程等8个项目建成投入使用，厦深铁路潮州段、仙洲岛堤围达标整治等11项工程开工建设，三百门电厂3、4号机组等项目前期工作扎实推进。

二、工业发展水平进一步提升

结构调整步伐加快。全市工业增加值208亿元，增长16.9%。其中，规模以上工业增加值108亿元，增长17.9%。工业规模化、适度重型化趋势更加明显。新增规模以上工业企业91家，累计850家;新增产值超亿元企业9家，累计75家。规模以上重化工业完成增加值44亿元，增长25%。能源、石化等临港工业发展加快，临港工业增加值12.81亿元，增长41.2%。轻重工业比例由上年的62：38调整为59：41。

自主创新能力增强。新认定省级以上高新技术企业40家，累计149家，数量居全省第9位。其中，国家高新技术企业2家，累计10家。新增省级工程中心3家，累计15家；省级企业技术中心4家，累计8家。省级以上科技项目立项83项，其中粤港澳关键领域重点突破项目中标6项，占粤东地区中标总量的60%。实施技术改造和创新项目92个，完成技改投资10.5亿元，增长14.1%。陶瓷、食品、不锈钢3个国家、省质量检验检测中心建设进展顺利。全年高新技术产品产值约130亿元，增长30%。

质量工作成效明显。产品质量和食品安全专项整治达到预期目标。技术标准化工作取得新突破，受国家有关部门委托，承担5个工业产品国家标准的制修订工作，负责组建“国家家用电器标准化技术委员会水族箱工作组”。有82个产品获得采标认可，数量居全省第4位，累计有290家企业295个产品采用国际标准和国外先进标准组织生产。

品牌战略成果丰硕。新增中国驰名商标1件、中国名牌产品4项、国家免检产品5项、省名牌产品27项、省著名商标23件，累计获得省级以上名牌名标207项。其中，中国名牌产品18项，数量居全省第5位。有21家企业获得“广东省出口名牌”称号，数量占全省的21%。潮安县凤塘镇被授予“中国陶瓷重镇”称号，庵埠镇和黄冈镇被认定为省火炬计划“果蔬加工特色产业基地”和“水族机电特色产业基地”。

三、“三农”工作进一步加强

农业发展水平有新提高。落实粮食生产扶持政策，全年粮食种植面积101.52万亩、总产44.1万吨，完成省下达计划。茶叶、水果、花卉、桑蚕等特色农业生产规模不断扩大，效益进一步提高。在“2007中国（广州）国际茶叶博览会”上，我市有26个茶叶产品获奖，数量占全省获奖总数的45.6%。渔业产业健康发展，深水抗风浪网箱养殖得到推广，水产养殖产量增长12.3%。农业产业化标准化继续推进，在25家市级以上农业龙头企业和5家市级重点农产品流通企业的带动下，全市8万多农户实现增产增收；省级凤凰农业现代化示范区、市级5个农业园区和15个生产基地的示范带动作用进一步增强。全年新认定无公害产品、绿色食品、有机食品19项，累计108项。

新农村建设不断推进。“以工促农、以城带乡”能力增强，农村生产生活条件日益改善。新农村示范村建设扎实推进，成效明显。农村富余劳动力技能培训工作进一步加强，近3万富余劳动力得到有效转移。扶贫开发成效显著，农民从各项惠农政策中得到的实惠约4.37亿元。

“一保五难”较好解决。全市农村3.8万名低收入群众全部纳入最低生活保障。农村安居工程完成危房改造3390户，累计13781户，超额完成省下达的任务。农村15.28万人饮水特别困难问题全部解决。镇通村公路硬底化建设步伐加快，有89.1%的行政村实现镇通村公路硬底化，可望提前一年实现省提出的目标。农村义务教育政策得到落实，有29.15万人接受免费教育。

四、内外源经济进一步发展

外经贸出现新变化。受出口退税政策调整等诸多因素影响，陶瓷、服装等传统出口产品进入结构性调整期。全市外贸出口总额17.7亿美元，下降1.8%。但技术含量和附加值较高的机电产品出口总值2.78亿美元，增长24.2%。招商引资水平有新提高。新批利用外资项目37个，合同利用外资1.34亿美元，增长11.2%；实际利用外资0.82亿美元，增长21%。

国内市场有效拓展。积极组织企业参加各类经贸活动，“潮货”知名度和市场占有率进一步提高。全市规模以上工业销售产值432.71亿元，增长22%，其中内销增长28%。“万村千乡”市场工程试点顺利，有3家配送中心和172家农家店通过省的资格验收。全社会消费品零售总额141.5亿元，增长19.8%。

文化旅游业加快发展。旅游宣传推介力度加大，各具特色的文化景点、自然生态景区逐步得到开发。市第二届文化旅游节的成功举办，广济桥等文物景点的修复开放，使潮州文化旅游吸引力迅速增强，我市成为省内最受欢迎的旅游目的地之一。全年接待海内外游客216.4万人次，旅游收入31.1亿元，分别增长14%和13.1%。

五、可持续发展能力进一步提高

国土资源管理力度加大。调整土地利用总体规划，优化了建设用地布局。开展“土地执法百日行动”等专项整治，查处一批违法占地用地行为。合理统筹使用存量土地，土地储备数量增加。工业园区建设力度加大，集约利用水平有所提高。深圳南山（潮州）产业转移工业园获得省认定，市经济开发区产业转移园区建设前期准备工作基本完成。整理开发耕地2210多亩，实现耕地占补平衡。瓷土、砂石等矿产资源管理工作加强。

生态环境建设不断推进。大力保护、培育和合理利用森林资源，积极防治水土流失和地质灾害，生态状况不断改善。潮安县创建林业生态县通过省的验收。认真抓好韩江、黄冈河污染整治等环境保护工作，建成镇级人工湿地污水处理系统3个，关闭污染严重的企业16家，化学需氧量排放总量削减5.88%。我市在国家城市综合整治定量考核中名列全省第5位。

节能降耗工作扎实有效。积极推进节能、节材以及资源综合利用工作，一批节能技术改造项目被国家发改委列入计划，四通绿环公司被省列为发展循环经济试点单位，兴业公司的陶瓷窑炉节能降耗技术在全省推广应用，全市单位GDP能耗下降3.43%。县级供电企业体制改革稳妥推进。

城市规划建设管理加强。《潮州市城市总体规划》等一批规划加紧修编，城乡规划体系不断完善。城市管理工作加强，环境卫生质量明显改善。金山大桥建成通车，中山路、绿榕南路等城市道路，以及市博物馆、文化艺术中心、牌坊街等公共设施建设进展顺利。桥东水厂扩建工程完成，城市供水范围不断扩大。

城市环境进一步改善。围绕提升城市品位这一中心，大力推进园林绿化景观建设，城市绿化、美化、净化和亮化水平提高，城市建成区绿地率36.3%、绿化覆盖率40.6%、人均公共绿地面积10.2平方米，创建“国家园林城市”顺利通过评审，被国家建设部命名为“国家园林城市”。韩江潮州河段环境整治与滨江景观建设工程，被国家建设部授予“中国人居环境范例奖”。

六、和谐社会建设进一步推进

文教卫体事业不断发展。公共文化服务体系建设成效显著，广播电视“村村通”工程扎实推进。潮州工夫茶等7个项目被列入第二批省级非物质文化遗产名录。农村校舍危房改造投入资金6488万元，维修加固校舍134幢，新建校舍114幢，全面实现省要求的预期目标。市卫生学校新校区二期工程竣工并投入使用，韩山师院陶瓷学院、市技工学校和市职业技术学校新校区建设进展顺利，市金山中学通过国家级示范性高中验收。高考本科入围率和录取率均超过全省平均水平。卫生事业有新发展，市中心医院内科大楼等重点卫生设施建设加快，社区卫生服务机构建设逐步完善。无偿献血工作成效显著，临床用血基本得到保障。新型农村合作医疗继续发展，参合率达到98%。食品监管力度加大，用药安全工作加强。人口计生工作落实，计生升类工作成效显著。群众性体育设施不断改善，形式多样的体育活动蓬勃开展，首届龙舟赛在海内外产生了积极影响。

劳动保障工作继续加强。全市城镇新增就业20166人，城镇登记失业率2.43%。启动生育保险、城镇职工住院医疗保险，开展被征地农民养老保障试点工作。养老、失业、工伤和医疗保险参保人数分别比上年增长8%、7.2%、7.6%和164.7%。各项社保基金征收6.69亿元，增长39.5%，当年收支结余1.99亿元。企业退休职工人均每月增加养老金116元。城乡居民最低生活保障标准提高。第二批“千间敬老福星工程”7所敬老院全部建成投入使用。

居民生活质量有新提高。市区居民人均可支配收入10391元，增长8.5%；消费性支出9314元，增长6.7%。农民人均纯收入4599元，增长6.5%。公共财政体系进一步健全，公共财政支出23.88亿元，增长22.4%。市区“双困户”住房租赁补贴制度落实，住房解困工作在全省会议上作了经验介绍。普惠型公益事业得到发展。

民主法制建设全面加强。自觉接受人大法律监督和政协民主监督，人大代表建议、政协委员提案办复满意或基本满意率100%。坚持依法行政，新制订规范性文件15个，依法解决社会矛盾的意识和能力增强。全面实施《行政复议法实施条例》，行政复议工作进一步加强。

行政服务管理水平提高。第三批行政审批制度改革完成。行政服务工作进一步加强，为民服务全程代理制全面铺开。政务公开继续推进，电子监察系统投入使用，行政效能监管不断深化。“五五”普法进展顺利。

社会保持稳定和谐。治安防控体系进一步健全，治安重点区域和突出问题得到有效整治。应对和处置突发公共事件机制逐步完善，安全生产形势稳定，信访工作得到加强。精神文明建设不断推进，“六好”平安和谐社区创建活动积极开展，连续四届荣获“广东省文明城市”称号。

与此同时，妇女儿童、民族宗教、双拥、人事、统计、审计、外事侨务、对台、老龄、气象、人防、地震、档案、地方志和残疾人等各项工作有新发展。

各位代表，过去一年所取得的成绩，是全市人民共同努力的结果，是驻潮部队、上级驻潮单位和各民主党派、人民团体大力支持的结果，也是港澳台同胞、海外侨胞，以及关心、支持和参与潮州建设事业的各阶层人士齐心协力的结果。在此，我代表市人民政府表示崇高的敬意和衷心的感谢！

我们也清醒地看到，经济社会发展中还存在一些困难和问题。一是经济总量不大，经济发展方式比较粗放。二是产业结构调整优化步伐不快，力度不够。三是受信贷资金等多种因素制约，固定资产投资乏力。四是建设用地供求矛盾突出，乱占乱用土地现象较为严重，工业不进园区的不良后果逐步显现。五是招商引资的新思路、新办法不多，成效不明显。六是节能减排压力大，治污减排力度不够，设施建设滞后。七是社会保障仍未能适应发展新要求，不少企业对劳动保障的认识还没有到位。八是政府工作还需进一步加强和改进，部分职能部门的创新意识、服务意识、大局意识有待提高。存在的困难和问题，我们必须在新的一年采取措施加以克服和解决。

2008年工作安排

2008年是全面贯彻落实党的十七大和省委十届二次全会精神的重要一年，也是实现“三年打基础、五年大变化、十年大发展”目标、奋力建设“一名城两基地”的关键一年。政府工作的指导思想是：高举中国特色社会主义伟大旗帜，以邓小平理论和“三个代表”重要思想为指导，全面贯彻落实科学发展观，按照“发展为先、民生为大、和谐为要、落实为重”的要求，团结带领全市人民继续解放思想，坚持改革开放，创新发展理念，转变发展方式，破解发展难题，提高发展质量，不断增强综合实力和竞争力，使全市人民在共建和谐中共享发展成果，为广东争当实践科学发展观排头兵作出应有贡献。

综观今年国内外形势，我市发展既面临着难得的新机遇，也面临着严峻的新挑战。从国际看，世界经济仍将保持增长，国际产业转移趋势增强，这有利于我们以开放促发展。从国内看，党的十七大为经济社会发展指明方向，科学发展观的贯彻落实，为欠发达地区推进发展创造了有利条件，奥运会的举办带来了经济增长的乐观预期，国内经济将继续保持又好又快发展。省委、省政府提出以新一轮的思想大解放，促进广东新一轮的大发展，为全省发展注入新的活力，也为潮州加快发展提供了良好契机。从我市看，经过多年来的努力和积累，发展环境大为改善，经济活力和竞争力不断提升；市开发区产业转移工业园和潮州港经济区的开发建设，为产业的调整优化创造了条件，为争取跨越式发展提供了空间；一批重点项目的建设和大型企业的落户，产生了较大的支撑和带动作用。但是，世界经济增长的不稳定性和贸易壁垒的增多，国内宏观调控力度的加大，发展成本的全面攀升，也使今年的发展面临诸多困难和压力。综合考虑各种因素，今年经济社会发展的主要预期目标是：生产总值增长15%，单位生产总值能耗下降3.5%，化学需氧量排放总量削减6.2%。地方财政一般预算收入增长18%，全社会固定资产投资增长15%，外贸出口总额增长5%，全社会消费品零售总额增长19%，居民消费价格涨幅控制在4%以下，人口自然增长率控制在5.97‰以下，城镇登记失业率控制在3%以下，市区居民人均可支配收入增长8%，农村居民人均纯收入增长5%。

为实现上述目标，要坚持把解放思想贯穿于落实科学发展观、推动潮州经济社会又好又快发展的全过程，着力转变发展观念，破解发展难题。进一步破除小富则安思想，强化奋发图强意识；破除因循守旧思想，强化改革创新意识；破除无所作为思想，强化干事创业意识；破除急功近利思想，强化持续发展意识；破除片面狭隘思想，强化顾全大局意识。通过继续解放思想，树立科学发展新观念，形成科学发展新思路，拿出科学发展新办法，解决科学发展新问题，以新一轮思想大解放推动新一轮大发展。

按照上述指导思想、主要目标和工作要求，要全力推进四方面工作。

一、转变经济发展方式，促进又好又快发展

推动经济又好又快发展，必须抓住转变经济发展方式这个关键，突出抓好以下工作。

（一）做大做强特色产业

坚持创新驱动。推进建立支柱产业关键共性技术创新中心、产业集群技术服务平台、骨干企业技术中心“三位一体”的产业技术创新体系。以建设陶瓷研究院为重点，抓好“中国瓷都创新基地”建设。加快发展专业镇和产业集群，完善和培育陶瓷、服装、食品、水族器材、不锈钢制品、塑料制鞋、印刷包装等特色产业基地。认真落实自主创新促进政策，用好省产业发展引导资金，扶持发展一批创新型企业。实施各类科技计划和技术改造项目，改造提升传统产业，大力发展高新技术产业。加强知识产权保护，为技术创新提供服务。积极引导企业创新经营观念和管理模式，推进建立现代企业制度，促进企业做大做强。

突出品牌带动。完善品牌培育机制，重点培育中国名牌产品、中国驰名商标和中国出口名牌，引导支持名牌名标企业争创世界名牌。争取水族器材列入中国名牌评价目录，扩大凉果、日用陶瓷国家级品牌拥有量。发展枫溪陶瓷国际区域品牌，扶持塑料工艺鞋类等产业争创区域品牌。落实《关于鼓励和促进企业上市的意见》，促进三环、雅士利、华丰等一批企业尽快上市，着力培育更多上市后备企业，使上市企业成为重要的区域品牌。重视品牌经营保护，进一步把品牌优势转化为市场优势和产业优势，以品牌带动产业发展，以品牌促进效益提高。

重视规范推动。加强技术质量标准化建设，全面推进食品质量安全市场准入制度、强制性产品认证制度。引导企业按照国内国际标准组织生产经营，解决卫生洁具、不锈钢制品等行业性质量问题。积极参与国家、行业标准的制修订。建设好陶瓷、食品、不锈钢等国家和省级检测中心。继续加强产品质量和食品安全工作，巩固发展专项整治成果。

抓好融资拉动。引导企业完善内部管理，加快企业信用体系和信用担保体系建设，营造良好的信贷环境。积极争取上级金融机构的信贷支持，发挥金融的服务和支持作用。大力推进股份制和股份合作制改造，鼓励支持企业通过上市、联手申报发行企业债券等形式，进一步拓展融资渠道。鼓励支持创办担保公司，有效解决中小企业贷款难的问题。

（二）大力发展港口经济

规划引导港口建设发展。做好国道324线碧岗至潮州港支线、港区供水工程和港区铁路支线的规划建设。严格执行《潮州港经济区总体规划》，编制重化工业园区、现代综合物流园区和科工贸开发园区控制性详细规划，优化港区产业布局。规划发展生产配套型和生活服务型等产业，加紧筹备申报潮州港保税物流中心和保税仓，推动转口贸易和加工贸易发展，大力培育港口水运市场，加快将潮州港建设成为集工业、商贸、仓储、流通于一体的港口经济区。按照《潮州港总体规划》，科学合理使用港口岸线。加大力度推进航道、码头建设，进一步完善口岸各项配套设施。争取大唐电厂5万吨级专用煤码头通过验收成为对外开放码头。

加强项目建设和招商。积极推动潮州港亚太通用码头、海峻石化项目、华丰二期工程、大唐电厂3、4号机组和中远石油储备项目建设。加强联系沟通，促进已签约的风能发电项目尽快投资建设，争取西气东输粤东支线终点门站落户港区。进一步做好宣传推介工作，吸引更多大型企业落户潮州港经济区，重点引进一批带动能力强、有利于延伸能源石化产业链条的项目，推动临港制造业和加工业发展。加快编制原油炼化以及原油、成品油码头等项目建议书，为招商引资创造条件。

（三）切实推进“三农”工作

加强农业农村基础建设。加快韩江南北堤南堤段、黄冈河大堤等城乡防灾减灾工程建设。抓紧做好沟尾溪、潮安内洋、湘桥区河内湖“三大涝区”整治前期工作。抓好村镇规划，加快中心镇和专业镇建设，推进新村建设和旧村改造，积极创建生态文明村。进一步构建城乡衔接的公共交通、供水供电体系。

推动现代农业提质增效。稳定粮食种植面积。提升茶叶、水果、水产、畜牧、花卉等特色农业发展水平，扩大无公害、绿色、有机农产品生产规模。加强农业标准化示范区和农业现代化示范区建设，建立“一乡一品”或“数乡一品”商品化基地。壮大农业龙头企业和流通组织，延长农业产业链。抓好动物疫病防控工作，推进禽畜产品安全设施建设。

加快现代渔业发展。大力发展深水抗风浪网箱养殖，进一步提升海水养殖水平。推进渔业标准化生产，建设一批现代标准鱼虾塘。加快沿海人工鱼礁建设步伐，完善以三百门渔港为重点的渔业基础设施。建设柘林湾水产品加工园区和饶平县粤东水产品物流中心。积极做好渔民转产转业工作。

落实各项支农惠农政策。及时发放各种涉农补贴资金。进一步解决农村“一保五难”问题，逐步提高农村交通、医疗、文化、教育等基本公共服务水平。探索建立“村企合作共建新农村”模式，增加新农村建设示范点，推进社会主义新农村建设。

（四）加快发展文化旅游业

加强古城区的保护与展示。围绕打造“国内有重要影响力的历史文化名城”这一目标，加紧完成名城保护规划和古城区控制性详细规划修编工作，切实加强古城区保护，全面展示古城风貌。充实广济桥的文化内涵，宣传好、经营好、管理好广济桥，使之更具吸引力和影响力；加快牌坊街的修复工作，做好牌坊街的营销策划，使之成为古城文化旅游区的新亮点。进一步整合古城文化旅游资源，做好己略黄公祠、笔架山宋窑等重点文物修复和太平桥开挖前期工作，筹划建设华侨博物馆（暂名）。规划建设传统工艺品街区、美食街区，配套建设木雕、潮绣等参观点，不断提升和丰富古城文化旅游区内涵。

扩大潮州文化旅游影响力。突出广济桥和牌坊街的龙头作用，充分发挥“中国瓷都”、“中国优秀旅游城市”、“国家园林城市”等城市品牌的综合效应，全方位加强对外宣传推介。创新宣传方式，拓宽宣传途径，完善旅游咨讯系统，推进旅游宣传信息化建设，把潮州旅游推向世界。协作做好粤东和海峡西岸旅游规划，着力突出潮州文化旅游地位。积极参加各种旅游推介活动，加强区域旅游宣传促销合作。

进一步改善旅游发展环境。配套完善古城区旅游服务设施和功能。成立古城区文物管理机构，提高文物景区的管理和经营水平。加强旅游硬件设施建设，提高旅游接待能力。抓好景区景点标准化建设管理，争创4A级景区2-3个。加快星级酒店建设，争取新发展高星级酒店1-2家。创新旅游发展机制，支持民资民力投资旅游业。发展乡村生态游和工业文化游，加快凤凰山、凤翔峡、道韵楼等景区景点建设。加强行业监管，规范旅游企业经营行为。重视人才队伍建设，着力提高旅游从业人员的素质。

（五）实施市场多元化战略

积极促进外贸出口。主动适应国家政策调整，优化出口结构，加快创新型出口基地建设，扩大机电产品、高新技术产品和加工贸易出口。加强出口监管，坚决查处高值低报等违法违规行为。发挥行业协会作用，积极应对贸易摩擦。加强行业自律，遏制低价竞销。强化以质取胜战略，提升跨越贸易技术壁垒能力。办好第二届中国（广州）国际陶瓷展览会，组织参加各类国际性经贸交流活动。完善出口激励机制，落实出口鼓励措施。扶持发展外贸出口龙头企业。优化通关环境，千方百计扩大出口。

大力开拓国内市场。研究探索开拓国内市场的办法和途径，推广市场开拓的成功经验，促进企业完善营销网络。引导企业准确把握国内市场需求，及时调整优化产品结构，抢占国内市场。组织参加“山洽会”、“中博会”等各类经贸活动。采取“政府搭台、企业唱戏”方式，争取在国内辐射力较强的中心城市举办大型经贸活动，努力扩大潮货在国内市场的份额。

提高招商引资水平。创新利用外资方式，切实把文化、产业、侨乡、港口等优势转化为招商引资优势。组织参加省首届“侨博会”，发挥招商引资顾问作用，大力实施“乡贤回归”工程，进一步拓宽招商引资渠道。鼓励现有外资企业增资扩产，扩大利用外资规模。积极参与泛珠三角等区域合作，密切与海峡西岸经济区的经贸关系。推动招商引资向招商选资转变，提高利用外资质量和水平。

完善现代市场体系。推进现代流通方式发展，努力壮大现代流通产业。发展各类生产要素市场，鼓励发展物流、金融、信息等现代服务业，提升商贸流通等传统服务业发展水平。组织实施全市商业网点规划建设，大力发展茶叶、陶瓷等特色产品市场和旅游品市场。推进“万村千乡”市场工程试点工作，拓宽农村商品流通渠道。

二、抓好环境资源保护，增强可持续发展能力

实现可持续发展，必须突出抓好节能降耗、资源节约集约利用和环境改善工作。

（一）推进节能环保工作

加强环境生态建设。坚持把生态建设与环境保护贯穿于发展的各个领域，努力实现经济增长、污染下降、生态改善。推进沿海防护林、水源涵养林等生态工程建设。做好韩江和黄冈河综合治理，切实保护饮用水源。加强农村环境保护，严防污染企业和城市污染物向农村扩散。推进环境保护基础设施建设，提高治污保洁能力。加快建设市污染源监控中心，提高环境监测水平。加强湿地、海洋、地质环境保护。认真做好第一次全国污染源普查。启动创建国家环保模范城市工作。

全力抓好节能减排。完善并严格执行能耗和环保标准，限制高污染、高能耗行业发展，逐步淘汰落后生产能力。推广节能降耗新技术，推进循环经济发展。加强对重点行业、重点企业的执法和监控，引导企业实施节能环保技术改造，促进陶瓷、不锈钢、电力等重点产业的资源节约和综合利用。改造市第一污水处理厂，做好桥东污水处理厂和排污管网建设前期工作，加快潮安、饶平县城和4个镇级污水处理设施建设。严格落实节能环保责任制和考核办法，确保完成省下达的约束性节能减排任务。

（二）加强资源节约集约利用

加强国土资源管理。落实土地和耕地管理责任，加强耕地和林地保护。严格执行土地利用总体规划，用好土地利用年度计划。加大土地开垦整理力度，确保耕地占补平衡。处置盘活闲置土地，做好土地储备工作。建立节约集约用地评价体系，落实工业用地招投标制度，推进低效用地二次开发，提高土地使用效率和投资强度。加强土地执法监察，严厉查处土地违法违规行为。做好新一轮土地利用总体规划修编工作，优化土地布局和结构。积极配合做好主体功能区规划编制工作，合理界定国土空间开发类别。加强矿产资源管理，坚决查处乱采滥挖违法行为，进一步防治地质灾害。

加快工业园区建设。完善深圳南山（潮州）产业转移工业园配套设施建设，督促已取得用地的项目加快建设，尽快形成生产力。加紧推进潮州经济开发区产业转移园区建设，为拓展产业发展空间、承接产业转移、引导工业进入园区、促进产业集聚创造条件。积极探索新形势下征地租地的有效办法和途径，合理规划建设各类工业园区，解决工业企业无序发展、遍地开花、村村冒烟和污染环境问题。

（三）不断优化发展环境

推动投资稳步增长。深化投融资体制改革，引导资本投向重化工业、装备制造业、环保产业、高新技术产业、基础设施等领域。制订实施《重点项目管理办法》、《政府投资项目管理暂行办法》，实行政府投资非经营性项目代建制，提高项目建设管理水平。加强新开工项目管理，推动42个年度重点项目顺利实施。完善征地拆迁和融资办法，努力解决重点项目用地难和信贷难问题。

加强城市规划建设管理。尽快完成新一轮城市总体规划的修编和各层次、各专项规划的编制，加强城市规划的实施管理，推动城区跨江发展，扩大城市发展架构，拓展城市发展空间。完善市政基础设施，抓紧做好潮州大桥（暂名）、银槐北路、外环北路等项目的规划设计建设，加快绿榕南路建设步伐。加强城市管理，巩固提高创园成果。发展城市公交，合理组织城市交通。

实施通达通畅工程。积极配合推进厦深铁路潮州段建设。加快省道222线三饶至浮山路段、省道334线上善至三饶路段的改造，努力提高公路等级标准和管养水平。推进县道沙土路和周边出口路建设。全面完成行政村通村公路硬底化建设任务。

三、着力改善民生，促进社会和谐发展

实现社会和谐稳定、百姓安居乐业，是政府的重要职责。要采取更加有力措施，确保人民群众学有所教、劳有所得、病有所医、老有所养、住有所居，促进改革发展和社会文明进步。

（一）加快社会事业发展

优先发展教育事业。加强基础教育，落实城乡免费义务教育政策，提升初中阶段办学水平。加快普通高中建设，扩大办学规模。大力发展职业教育，争取市职业技术学校新校区和韩山师院陶瓷学院在下半年投入使用。加快学校新装备和信息化建设。

推动文化体育发展。加强文物和非物质文化遗产保护。加快文化艺术馆、博物馆、图书馆和档案馆等重点文化工程建设，推进“农家书屋”、广播电视“村村通”等工程，不断完善社区和乡村文化设施，进一步活跃群众文化生活。扶持发展文化产业。广泛开展全民健身运动，建设一批健身工程，改善群众健身条件。办好潮州市第九届体育节。

加强人口计生工作。完善和落实人口与计划生育层级动态管理责任制和利益导向机制。加强对流动人口的计生管理和服务工作。稳定低生育水平，提高出生人口素质。

加快医药卫生发展。搞好农村医疗卫生三级服务网络和社区卫生服务机构建设，提高公共卫生服务水平。加强卫生监督执法，做好疾病预防控制。推进实施新型农村合作医疗制度。加快城乡中医药服务体系和农村药品“两网”建设。

加强现代公民素质教育。大力弘扬“开放、务实、创新、奉献”的新时期潮州人精神，抓好社会道德教育，倡导科学健康文明生活方式。构建学习型社会，积极推进“六好”平安和谐社区建设，不断提高市民素质和社会文明程度。

加大双拥工作力度，创建新一届“广东省双拥模范城”。继续做好人才、民族宗教、广播电视、新闻出版、外事侨务、对台、人防、防震减灾、民兵预备役、气象、统计、档案、地方志等工作。

（二）努力做好民生工作

实施积极就业政策。加快构建城乡一体化就业服务体系，推进城乡统筹就业，争取城镇新增就业岗位2万个，帮助城镇“零就业家庭”、农村贫困家庭实现劳力非农就业。加强职业教育、职业培训，重点抓好农村劳动力转移和退役士兵职业技能培训，努力解决“招工难”与“就业难”结构性矛盾。进一步规范企业用工行为，切实保障劳动者合法权益。

完善社会保障制度。扩大养老保险覆盖面，提高企业缴费标准，增加企业退休人员养老待遇。加强城镇职工基本医疗保险，实施生育保险、城镇居民医疗保险。建立被征地农民养老保险制度。加大社会保险费征缴和基金监管力度。建立健全新型社会救助、优抚安置、社会福利等服务体系，发展慈善事业和残疾人事业，开展助贫、助困、助残、助学等活动，提高社会救助能力。

改善群众住房和饮水条件。加快经济适用房和解困房建设，积极实施农村泥砖房改造。依法和谐做好拆迁及管理工作。加快实施农村饮水安全工程，重点推进18宗省批准立项的工程建设。

保持价格总水平基本稳定。加强价格监管，做好粮、肉、油等重要商品供应。加强价格信息发布，稳定消费者预期心理。落实因基本生活必需品价格上涨对低收入群众的补助。

（三）促进改革和社会管理

深化重点领域改革。抓好政府采购制度、财政支出绩效评价、国库集中支付等改革，切实提高财政资金使用效益。实行新的财政体制，理顺市与区的分配关系，激发区级发展活力。坚持依法治税，促进税务工作实现新跨越。深化事业单位改革。加快国有、集体劣势企业依法依规退市，引导优势国有企业引进投资，妥善处理国企改制遗留问题。完善金融服务管理体系，优化金融生态环境。推动农村信用社改革发展，实现城信社和信托投资公司平稳退市。

加强和改进社会管理。强化社会治安综合治理，严厉打击各类违法犯罪活动，积极推进平安潮州建设。做好信访和社会稳定工作，引导群众按法定程序合理表达利益诉求。加强社会矛盾纠纷排查调处和预警体系建设，预防和妥善处置群体性事件。健全应急管理机制，完善各类应急预案，提高应对突发公共事件的综合能力。全面落实生产安全责任制，建立健全安全生产监管体系，杜绝重特大事故发生。

四、加强政府自身建设，提高公共服务水平

促进经济社会又好又快发展，人民期望殷切。要加强政府自身建设，全面提高行政管理和公共服务水平。

坚持与时俱进，建设创新政府。继续解放思想、转变观念，不断提高贯彻落实科学发展观的自觉性和主动性，进一步推进政府决策的科学化、民主化和法制化。加快知识更新，不断开阔视野，以世界眼光谋划科学发展。坚持从实际出发，从创新入手，努力形成干事创业的良好氛围。

转变政府职能，建设服务政府。创新政府管理服务的理念、机制和手段，加强社会管理和公共服务职能，注重统筹社会发展和解决民生问题。深化行政审批制度改革，不断完善政务公开的方式和载体。推进市、县行政服务中心建设，建立联合审批网络系统，全面完善为民服务全程代理制。积极发展社会事业，努力解决涉及群众切身利益的热点问题。增加社会保障、卫生、教育等投入，推进基本公共服务均等化。

严格依法行政，建设法治政府。按照法定权限和程序履行职责，强化政府法制监督。加强行政执法队伍管理，规范行政执法行为。继续推进“五五”普法工作。自觉接受人大和政协监督，认真办理人大代表建议、政协委员提案，关注社情民意。支持工会、共青团、妇联工作。加强基层民主政治建设，完成第四届村委会和第三届居委会的选举工作。进一步规范行政行为，强化部门之间的沟通协调，确保政令畅通。

树立务实作风，建设廉洁政府。坚持群众路线，强化群众观念，重视调查研究。大力弘扬艰苦奋斗、勤俭节约的光荣传统。加强廉洁从政教育，严格执行廉政责任制。强化行政监察和审计监督，坚决治理和纠正各种不正之风。大力倡导良好的文风会风，努力建设节约型机关。加强公务员队伍建设，全面提升公务员的服务意识和业务素质，树立执政为民良好形象。

各位代表，我市正处在经济社会发展全面转入科学发展轨道的关键时期。时代赋予使命，人民寄予厚望。让我们紧密团结在以胡锦涛同志为总书记的党中央周围，高举中国特色社会主义伟大旗帜，在中共潮州市委的领导下，群策群力谋发展，同心同德干事业，为实现科学发展、和谐发展而努力奋斗，为广东争当实践科学发展观排头兵作出积极贡献！
